# Effect of Real-time Patient-Centered Education Bundle on Administration of Venous Thromboembolism Prevention in Hospitalized Patients

**DOI:** 10.1001/jamanetworkopen.2018.4741

**Published:** 2018-11-16

**Authors:** Elliott R. Haut, Jonathan K. Aboagye, Dauryne L. Shaffer, Jiangxia Wang, Deborah B. Hobson, Gayane Yenokyan, Elizabeth A. Sugar, Peggy S. Kraus, Norma E. Farrow, Joseph K. Canner, Oluwafemi P. Owodunni, Katherine L. Florecki, Kristen L. W. Webster, Christine G. Holzmueller, Peter J. Pronovost, Michael B. Streiff, Brandyn D. Lau

**Affiliations:** 1 Department of Emergency Medicine, The Johns Hopkins University School of Medicine, Baltimore, Maryland; 2 Division of Acute Care Surgery, Department of Surgery, The Johns Hopkins University School of Medicine, Baltimore, Maryland; 3 Armstrong Institute for Patient Safety and Quality, Johns Hopkins Medicine, Baltimore, Maryland; 4 Johns Hopkins Surgery Center for Outcomes Research, The Johns Hopkins University School of Medicine, Baltimore, Maryland; 5 Department of Anesthesiology and Critical Care Medicine, The Johns Hopkins University School of Medicine, Baltimore, Maryland; 6 Department of Health Policy and Management, Johns Hopkins Bloomberg School of Public Health, Baltimore, Maryland; 7 Department of Surgery, The Johns Hopkins University School of Medicine, Baltimore, Maryland; 8 Department of Nursing, The Johns Hopkins Hospital, Baltimore, Maryland; 9 Department of Biostatistics, Johns Hopkins Bloomberg School of Public Health, Baltimore, Maryland; 10 Department of Pharmacy, The Johns Hopkins Hospital, Baltimore, Maryland; 11 Department of Surgery, Duke University, Durham, North Carolina; 12 Division of Hematology, Department of Medicine, The Johns Hopkins University School of Medicine, Baltimore, Maryland; 13 Russell H. Morgan Department of Radiology and Radiological Science, The Johns Hopkins University School of Medicine, Baltimore, Maryland; 14 Division of Health Sciences Informatics, The Johns Hopkins University School of Medicine, Baltimore, Maryland

## Abstract

**Importance:**

Numerous interventions have improved prescription of venous thromboembolism (VTE) prophylaxis; however, many prescribed doses are not administered to hospitalized patients, primarily owing to patient refusal.

**Objective:**

To evaluate a real-time, targeted, patient-centered education bundle intervention to reduce nonadministration of VTE prophylaxis.

**Design, Setting, and Participants:**

This nonrandomized controlled, preintervention-postintervention comparison trial included 19 652 patient visits on 16 units at The Johns Hopkins Hospital, Baltimore, Maryland, from April 1 through December 31, 2015. Data analysis was performed from June 1, 2016, through November 30, 2017, on an intention-to-treat basis.

**Interventions:**

Patients on 4 intervention units received a patient-centered education bundle if a dose of VTE prophylaxis medication was not administered. Patients on 12 control units received no intervention.

**Main Outcomes and Measures:**

Conditional odds of nonadministration of doses of VTE prophylaxis (primary outcome) before and after the intervention on control vs intervention units. Reasons for nonadministration (ie, patient refusal and other) and VTE event rates (secondary outcomes) were compared.

**Results:**

A total of 19 652 patient visits where at least 1 dose of VTE prophylaxis was prescribed were included (51.7% men; mean [SD] age, 55.6 [17.1] years). Preintervention and postintervention groups were relatively similar in age, sex, race, and medical or surgery unit. From the preintervention period to the postintervention period, on intervention units, the conditional odds of VTE prophylaxis nonadministration declined significantly (9.1% [95% CI, 5.2%-16.2%] vs 5.6% [95% CI, 3.1%-9.9%]; odds ratio [OR], 0.57; 95% CI, 0.48-0.67) compared with no change on control units (13.6% [95% CI, 9.8%-18.7%] vs 13.3% [95% CI, 9.6%-18.5%]; OR, 0.98; 95% CI, 0.91-1.07; *P* < .001 for interaction). The conditional odds of nonadministration owing to patient refusal decreased significantly on intervention units (5.9% [95% CI, 2.6%-13.6%] vs 3.4% [95% CI, 1.5%-7.8%]; OR, 0.53; 95% CI ,0.43-0.65) compared with no change on control units (8.7% [95% CI, 5.4%-14.0%] vs 8.5% [95% CI, 5.3%-13.8%]; OR, 0.98; 95% CI, 0.89-1.08; *P* < .001 for interaction). On intervention units, the conditional odds of nonadministration owing to reasons other than patient refusal decreased (2.3% [95% CI, 1.5%-3.4%] vs 1.7% [95% CI, 1.1%-2.6%]; OR, 0.74; 95% CI, 0.58-0.94), with no change on control units (3.4% [95% CI, 2.7%-4.4%] vs 3.3% [95% CI, 2.6%-4.2%]; OR, 0.98; 95% CI, 0.87-1.10; *P* = .04 for interaction). No differential effect occurred on medical vs surgical units (OR, 0.86; 95% CI, 0.60-1.23; *P* = .41 for interaction). There was no statistical difference in the proportion of VTE events among patients on intervention vs control units (0.30% vs 0.18%; OR, 0.60; 95% CI, 0.16-2.23).

**Conclusions and Relevance:**

In this study, a targeted patient-centered education bundle significantly reduced nonadministration of pharmacologic VTE prophylaxis in hospitalized patients. This novel strategy improves health care quality by leveraging electronic data to target interventions in real time for at-risk patients.

**Trial Registration:**

ClinicalTrials.gov Identifier: NCT02402881

## Key Points

**Question:**

Can a real-time, targeted, patient-centered education bundle reduce nonadministration of venous thromboembolism prophylaxis in hospitalized patients?

**Findings:**

In this controlled preintervention-postintervention comparison trial of 19 652 adult patients on medical and surgical units, nonadministration of venous thromboembolism prophylaxis significantly declined on units that received an intervention that combined an alert to a health educator about a missed dose of venous thromboembolism prophylaxis with patient education compared with control units.

**Meaning:**

Timely, targeted education significantly reduces nonadministration of VTE prophylaxis in hospitalized patients and improves health care quality by leveraging real-time data to target interventions for at-risk patients.

## Teaser

This pre-post intervention comparison study evaluates a real-time patient-centered education bundle intervention aimed to reduce nonadministration of venous thromboembolism prophylaxis among hospitalized patients in surgical and medical units.

## Introduction

Venous thromboembolism (VTE) remains a leading cause of preventable morbidity and mortality among hospitalized patients^1,2,3,4^ and poses an immense economic burden.^5,6^ Numerous successful interventions have been implemented in hospitals around the world to improve prescription of appropriate VTE prophylaxis.^7,8^ However, a substantial proportion of prescribed VTE prophylaxis doses are not administered.^9,10,11,12,13,14^ The leading cause of nonadministration of VTE prophylaxis medication is patient refusal,^9,10^ the causes of which have been explored through quantitative and qualitative studies.^15,16^

The Johns Hopkins Hospital VTE Collaborative has worked for more than a decade to improve evidence-based VTE prevention practices, seeking to eliminate preventable VTE^17^ by ensuring all patients are prescribed appropriate VTE prophylaxis and receive all prescribed doses.^18,19^ In this study, we aimed to evaluate the association of a patient-centered education bundle with the nonadministration of VTE prophylaxis to hospitalized patients. We hypothesized that implementation of this bundle to a targeted group of patients who missed doses of VTE prophylaxis while hospitalized would increase dose administration and lead to fewer VTE events.

## Methods

### Study Setting and Design

We conducted a controlled study using a preintervention-postintervention experimental design that compared a patient-centered education bundle intervention with no intervention for improving administration of pharmacologic VTE prophylaxis. The intervention was implemented from April 1 through December 31, 2015, at The Johns Hopkins Hospital, Baltimore, Maryland. The trial protocol is available in the Supplement. All 16 medical and surgical adult nursing units were included, and intensive care units were excluded. A convenience sample of 4 units (2 surgical and 2 medical) received the intervention. The remaining 12 units (5 surgical and 7 medical) served as control units and received no intervention. We performed a power calculation based on the number of patients and the very large number of doses historically prescribed to determine whether we would have sufficient power for this study.^14^ We collected data on prescribed and administered pharmacologic VTE prophylaxis from the hospital electronic medical record (eMAR); baseline data were retrospectively collected for the period of October 1, 2014, through December 31, 2015. The eMAR system required documentation of all prescribed prophylaxis doses as administered or nonadministered, including the reason for nonadministration (Supplement). Venous thromboembolism events were captured using the Agency for Healthcare Research and Quality patient safety indicator (PSI-12, Perioperative Pulmonary Embolism or Deep Vein Thrombosis Rate) from our administrative database.^20^ Race was classified based on the eMAR. Our blinded biostatistician team (J.W., G.Y., and E.A.S.) was not involved in outcome determination; analyses were conducted from June 1, 2016, through November 30, 2017, following the Transparent Reporting of Evaluations With Nonrandomized Designs (TREND) reporting guideline for nonrandomized controlled trials.^21^ The institutional review board of Johns Hopkins Medicine approved this study, and the Johns Hopkins Medicine institutional review board provided a waiver of consent for this study.

### Intervention

We built a novel real-time alert in the hospital eMAR system that paged and emailed 1 health educator (D.L.S.), who worked half of a full-time equivalent on the project, when a prescribed dose of pharmacologic VTE prophylaxis was not administered, as documented by the bedside nurse. Once alerted, the health educator engaged the bedside nurse to determine the cause of the nonadministered dose. If the patient had refused, the health educator visited the patient to deliver the patient education bundle, which described VTE, risks, and prevention. If patient refusal was not the reason for the nonadministration, the health educator delivered the intervention on VTE prophylaxis and the need to give all prescribed doses if no contraindication was found to the bedside nurse. If the prophylaxis dose was not administered owing to a prescriber-documented contraindication (without a concomitant hold or discontinuation order), the educator contacted the prescriber (eg, resident physician, nurse practitioner, or physician assistant) to remedy the situation. The duration of time (minutes) spent on each educational intervention was recorded. Although the study health educator was not present 24 hours per day 7 days per week, data were collected on all nonadministered doses on all intervention and control units. All analyses were performed on an intention-to-treat basis.

### Patient Education Bundle

The patient education bundle used 3 different methods to deliver information about VTE and the benefits of prevention. Patients could choose to receive 1 or more components of the bundle, which included (1) one-on-one, face-to-face educational discussion with a health educator; (2) a 2-page paper handout (available in 8 languages, including English^22^), and (3) a 10-minute patient educational video^22^ shown on a handheld tablet. We developed the bundle using a modified Delphi method to build consensus on the content and optimal delivery of VTE prevention information to hospitalized patients.^23^ Input was received from more than 400 stakeholders from 3 national blood clot organizations and our local The Johns Hopkins Hospital Patient and Family Advisory Council.^23^

### Statistical Analysis

Our primary outcome was the proportion of nonadministered doses of prescribed pharmacologic VTE prophylaxis. Secondary outcomes included the proportions of doses that were not administered owing to patient refusal or another documented reason and the odds of developing in-hospital VTE. Our primary hypothesis was evaluated by comparing rates of VTE prophylaxis nonadministration before (October 1, 2014, through March 31, 2015) and after (April 1 through December 31, 2015) the intervention. We compared this change on intervention vs control units during the same periods. We hypothesized a differential effect for medical vs surgery units and performed a prespecified analysis stratified by unit type. Patient demographic characteristics for the preintervention and postintervention periods were described by study arm.

We used 2-sample *t* tests with equal variance to compare age and χ^2^ tests to compare sex, race, and unit type. We used the nonparametric Wilcoxon rank sum tests to compare the number of dosages and length of hospital stay.

To compare nonadmistration of VTE prophylaxis by group and time, we used generalized linear mixed-effects models with random intercepts for unit and nurse to account for correlation within unit and nurse. Because multiple VTE prophylaxis doses were administered per patient across nurses and/or units, we used multiple outputation to reduce the levels of hierarchical structure to the unit and nurse level by randomly selecting 1 VTE prophylaxis dose per patient and reiterating the procedure 1000 times to bootstrap the 95% CIs and the *P* values for the comparisons.^24^ The models included group (intervention vs control), time (preintervention vs postintervention), and the 2-way interaction as the primary indicators. We performed an a priori stratified analysis for patients on medical vs surgical units and included the 3-way interaction term (intervention vs control groups, preintervention vs postintervention periods, and medical vs surgical units). For estimating conditional odds ratios (ORs) and their 95% CIs, the binomial family and a logit link were used; for estimating the conditional proportions, the Poisson family and a log link were used. Stratified (or subgroup) analyses (ie, medical and surgical units) were performed using the same models to assess the same outcomes. We present the conditional probability of missed doses by month for the 4 strata (medical or surgical unit; intervention or control group) to show the association over time.

Our biostatistician team (J.W., G.Y., and E.A.S.) was blinded to floor assignment arms. All comparisons were performed at the .05 level of statistical significance, with 2-tailed *P* values. In cases of missing demographic data, we performed manual medical record review.^25^ Statistical analyses were performed using Stata/MP software (version 14.1, Parallel Edition; StataCorp).

## Results

**Figure 1.**
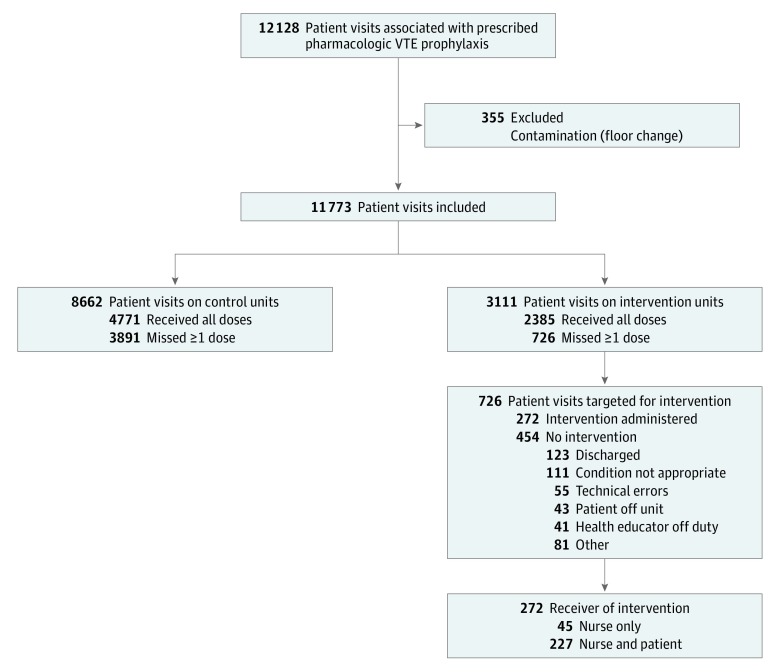
CONSORT Flow Diagram of Patients Receiving the Education Bundle Intervention Patient visits reflect prescribed pharmacologic venous thromboembolism (VTE) prophylaxis doses. A missed prophylaxis dose triggers an intervention; the bedside nurse and patients who refused at least 1 dose are delivered the patient education bundle. The bundle is only delivered once to participants.

A total of 19 652 patient visits were analyzed in which the patient was prescribed at least 1 dose of VTE prophylaxis medication during their hospitalization (51.7% men and 48.3% women; mean [SD] age, 55.6 [17.1] years). This sample included 7879 and 11 773 patient visits in the preintervention and postintervention periods, respectively. There were 5333 patient visits on intervention units and 14 319 on control units (Figure 1). We excluded 355 patient visits (2.9%) in which the patients were treated on an intervention and a control unit. Table 1 displays the demographic and clinical characteristics of the patient visits. Patients on control units were slightly older than those on intervention units (mean [SD], 56.3 [16.9] vs 53.9 [17.4] years; *P* < .001). Men accounted for a higher proportion of patients on control compared with intervention units (2970 [52.5%] vs 1069 [48.1%]; *P* < .001) in the baseline period; however, no evidence showed that the proportion changed within floor type between periods. The median number of prescribed VTE doses per patient visit was similar in the preintervention and postintervention periods on control (8 [interquartile range {IQR}, 4-14] vs 8 [IQR, 4-14]) and intervention (6 [IQR, 3-13] vs 7 [IQR, 3-13]) units (Table 1).

**Table 1.** Demographic and Clinical Characteristics of Preintervention and Postintervention Periods by Treatment Group

Variable	Intervention Group			Control Group			Preintervention Group Comparison, *P* Value
	Preintervention Period Visits (n = 2222)	Postintervention Period Visits (n = 3111)	*P* Value	Preintervention Period Visits (n = 5657)	Postintervention Period Visits (n = 8662)	*P* Value	
No. of unique patients	1736	2352	NA	4354	6543	NA	NA
No. of unique nurses	250	277	NA	742	832	NA	NA
Age, mean (SD), y^a^	53.9 (17.4)	54.6 (17.6)	.15	56.3 (16.9)	55.8 (16.9)	.08	.001
Sex, No. (%)^b^							
Male	1069 (48.1)	1524 (49.0)	.53	2970 (52.5)	4588 (53.0)	.61	<.001
Female	1153 (51.9)	1587 (51.0)		2687 (47.5)	4074 (47.0)		
Race, No. (%)^b^							
Black	941 (42.3)	1308 (42.0)	.29	2307 (40.8)	3469 (40.0)	.77	.40
White	1088 (49.0)	1533 (49.3)		2850 (50.4)	4432 (51.2)		
Asian	44 (2.0)	73 (2.3)		117 (2.1)	167 (1.9)		
Native American	9 (0.4)	4 (0.1)		12 (0.2)	14 (0.2)		
Other	140 (6.3)	193 (6.2)		371 (6.6)	580 (6.7)		
Unit type, No. (%)^b^							
Surgical	1155 (52.0)	1647 (52.9)	.49	2530 (44.7)	4076 (47.1)	.01	<.001
Medical	1067 (48.0)	1464 (47.1)		3127 (55.3)	4586 (52.9)		
No. of prescribed doses per patient visit							
Median (IQR)	6 (3-13)	7 (3-13)	.05	8 (4-14)	8 (4-14)	.006	<.001
Mean (SD)^c^	9.7 (10.6)	10.5 (13.5)		11.2 (12.7)	11.4 (12.9)		
Length of stay, d							
Median (IQR)	4 (2-7)	4 (2-7)	.02	4 (2-8)	5 (2-8)	.002	<.001
Mean (SD)^c^	5.8 (6.7)	6.3 (8.0)		6.8 (8.8)	7.2 (10.0)		

Abbreviations: IQR, interquartile range; NA, not applicable.

^a^ *P* values calculated using 2-sample *t* tests with equal variances.

^b^ *P* values calculated using χ^2^ tests.

^c^ *P* values calculated using Wilcoxon rank sum tests.

### Intervention Delivery

Among 726 patient visits on intervention units with at least 1 documented nonadministered dose of prescribed VTE prophylaxis, 272 interventions (37.5%) occurred. Of 272 interventions, 45 (16.5%) included nurses only and 227 (83.5%) included the nurse and patient. The health educator spent a median of 2 minutes (range, 1-25 minutes) with bedside nurses and a median of 10 minutes (range, 1-40 minutes) with patients.

The remaining 454 patient visits with at least 1 documented nonadministered dose did not receive the intervention. Of these patient visits, 123 (27.1%) were discharged before the intervention could be delivered, 111 (24.4%) had an order to discontinue the dose, 55 (12.1%) had technical errors resulting in a duplicate order, 41 (9.0%) occurred when the health educator was off duty, 43 (9.5%) were off the floor when the nurse educator visited, and 81 (17.8%) did not receive an intervention for other miscellaneous reasons (Figure 1). Of the 227 patients who received the patient education bundle, 87 (38.3%) chose only the paper handout, 2 (0.9%) chose only the video, 53 (23.3%) chose a combination of the video and paper handout, and 85 (37.4%) chose only the conversation with the nurse educator.

### VTE Prophylaxis Medication Administration

Overall, the conditional odds of nonadministration of a pharmacologic VTE prophylaxis dose decreased (OR, 0.88; 95% CI, 0.82-0.95) from the preintervention to postintervention periods (Table 2). The proportion of any nonadministered doses decreased from 9.1% (95% CI, 5.2%-16.2%) during the preintervention period to 5.6% (95% CI, 3.1%-9.9%) during the postintervention period, while there was no change in the proportion of any nonadministered doses during the pre- and postintervention periods (13.6% [95% CI, 9.8%-18.7%] vs 13.3% [95% CI, 9.6%-18.5%]) on control units. The conditional odds of nonadministration of VTE prophylaxis declined on intervention units (OR, 0.57; 95% CI, 0.48-0.67) and were unchanged on control units (OR, 0.98; 95% CI, 0.91-1.07). The decline on intervention units was significantly greater than on control units (OR, 0.58; 95% CI, 0.48-0.70; *P* < .001 for interaction).

**Table 2.** Proportion of Doses Missed Between Preintervention vs Postintervention by Treatment Group^a^

Period	Intervention	Control	OR Intervention vs Control (95% CI)	*P* Value^b^
**Any Missed Dose**				
Preintervention, % (95% CI)	9.1 (5.2-16.2)	13.6 (9.8-18.7)	0.63 (0.30-1.35)	.24
Postintervention, % (95% CI)	5.6 (3.1-9.9)	13.3 (9.6-18.5)	0.37 (0.17-0.79)	.01
OR Post- vs preintervention (95% CI)	0.57 (0.48-0.67)	0.98 (0.91-1.07)	NA	NA
*P* value^b^	<.001	.62	NA	NA
**Patient Refused Dose**				
Preintervention, % (95% CI)	5.9 (2.6-13.6)	8.7 (5.4-14.0)	0.66 (0.23-1.91)	.44
Postintervention, % (95% CI)	3.4 (1.5-7.8)	8.5 (5.3-13.8)	0.36 (0.12-1.03)	.06
OR Post- vs preintervention (95% CI)	0.53 (0.43-0.65)	0.98 (0.89-1.08)	NA	NA
*P* value^b^	<.001	.71	NA	NA
**Other Reasons Than Refusal**				
Preintervention, % (95% CI)	2.3 (1.5-3.4)	3.4 (2.7-4.4)	0.65 (0.39-1.06)	.09
Postintervention, % (95% CI)	1.7 (1.1-2.6)	3.3 (2.6-4.2)	0.49 (0.30-0.81)	.01
OR Post- vs preintervention (95% CI)	0.74 (0.58-0.94)	0.98 (0.87-1.10)	NA	NA
*P* value^b^	.01	.69	NA	NA

Abbreviations: NA, not applicable; OR, odds ratio.

^a^ Two-way interactions were performed including preintervention vs postintervention period and control vs intervention units (OR for any missed dose, 0.58 [95% CI, 0.48-0.70; *P* ≤ .001]; OR for patient refusal, 0.54 [95% CI, 0.43-0.68; *P* ≤ .001]; OR for other reasons than refusal, 0.76 [95% CI, 0.58-0.98; *P* = .04]).

^b^ Calculated using multiple outputation of the generalized linear mixed-effects models with the binomial family and a logit link.

### Reason for VTE Prophylaxis Nonadministration

Patient refusal was the most common documented reason for medication nonadministration (Table 2). Overall, the conditional odds of a refused dose declined (OR, 0.87; 95% CI, 0.80-0.95) from the preintervention to postintervention periods. On intervention units, patient refusal was 5.9% (95% CI, 2.6%-13.6%) during the preintervention period and 3.4% (95% CI, 1.5%-7.8%) during the postintervention period (*P* < .001), and the odds of refusing a dose decreased from the preintervention to postintervention periods (OR, 0.53; 95%, CI, 0.43-0.65). In contrast, there was no difference in the proportion of refusing a dose (8.7% [95% CI, 5.4%-14.0%] vs 8.5% [95% CI, 5.3%-13.8%]) on control units, and the odds of patient dose refusal were unchanged on control units (OR, 0.98; 95% CI, 0.89-1.08). The decline in dose refusal on intervention units was significantly greater than on control units (OR, 0.54; 95% CI, 0.43-0.68; *P* < .001 for interaction). The overall conditional proportion of doses not administered for reasons other than patient refusal was 3.1% in the preintervention period compared with 2.8% in the postintervention period (OR 0.93, 95% CI 0.83-1.04). On intervention units, the proportion of nonadministered doses for other reasons decreased from 2.3% (95% CI, 1.5%-3.4%) in the preintervention period to 1.7% (95% CI, 1.1%-2.6%) in the postintervention period (OR, 0.74; 95% CI, 0.58-0.94). On control units, this proportion was unchanged (3.4% [95% CI, 2.7%-4.4%] vs 3.3% [95% CI, 2.6%-4.2%]; OR, 0.98; 95% CI, 0.87-1.10). The reduction in doses missed for reasons other than refusal was significantly greater on intervention vs control units (OR, 0.76; 95% CI, 0.58-0.98; *P* = .04 for interaction).

### Stratified Analysis by Unit Type

**Figure 2.**
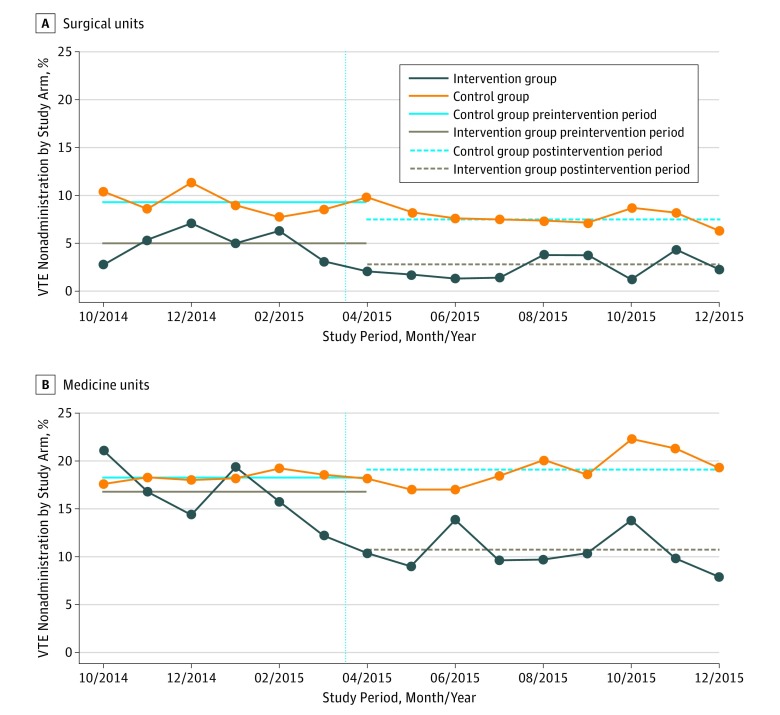
Conditional Proportion of Missed Doses of Venous Thromboembolism (VTE) Prophylaxis for Intervention and Control Arms on Medical and Surgical Units Data are stratified by month.

A prespecified subgroup analysis demonstrated that medical units had significantly higher rates of nonadministration than surgical units for the 4 comparisons. Before any intervention began, nonadministration was significantly lower on surgical floors in the intervention group (5.0% surgical vs 16.8% medical; *P* < .001) and the control groups (9.3% surgical vs 18.3% medical; *P* < .001) (Table 3). Intervention units had significantly lower rates of nonadministration than control units stratified by study arm and period (Figure 2).

**Table 3.** Subgroup Analysis by Unit Type on the Proportion of Prescribed Venous Thromboembolism Prophylaxis Medication Doses Missed^a^

Intervention Period	Surgical Units				Medical Units			
	Intervention Group	Control Group	OR Intervention vs Control (95% CI)	*P* Value	Intervention Group	Control Group	OR Intervention vs Control (95% CI)	*P* Value
**Any Missed Dose**								
Preintervention, % (95% CI)	5.0 (3.1-8.2)	9.3 (6.8-12.6)	0.50 (0.26-0.98)	.04	16.8 (10.3-27.2)	18.3 (14.1-23.8)	0.89 (0.47-1.69)	.72
Postintervention, % (95% CI)	2.8 (1.7-4.6)	8.0 (5.9-10.8)	0.32 (0.16-0.63)	.001	10.7 (6.6-17.3)	19.1 (14.8-24.7)	0.49 (0.26-0.92)	.03
OR postintervention vs preintervention (95% CI)	0.54 (0.42-0.69)	0.84 (0.74-0.95)	NA	NA	0.58 (0.47-0.72)	1.06 (0.96-1.18)	NA	NA
*P* value^b^	<.001	.01	NA	NA	<.001	.24	NA	NA
**Patient Refusal**								
Preintervention, % (95% CI)	2.5 (1.3-4.9)	4.6 (3.0-6.9)	0.52 (0.22-1.25)	.15	14.1 (7.6-26.4)	14.5 (10.4-20.3)	0.96 (0.44-2.13)	.92
Postintervention, % (95% CI)	1.3 (0.6-2.6)	3.7 (2.5-5.5)	0.34 (0.14-0.81)	.02	8.3 (4.4-15.4)	15.1 (10.8-21.0)	0.49 (0.22-1.08)	.08
OR postintervention vs preintervention (95% CI)	0.50 (0.34-0.75)	0.79 (0.66-0.93)	NA	NA	0.54 (0.43-0.68)	1.05 (0.94-1.18)	NA	NA
*P* value^b^	<.001	.01	NA	NA	<.001	.36	NA	NA
**Other Reasons Than Refusal**								
Preintervention, % (95% CI)	2.3 (1.3-3.9)	3.8 (2.7-5.5)	0.56 (0.28-1.11)	.10	2.2 (1.2-4.0)	3.1 (2.3-4.2)	0.71 (0.35-1.43)	.34
Postintervention, % (95% CI)	1.3 (0.8-2.4)	3.5 (2.5-5.0)	0.37 (0.18-0.74)	.01	2.1 (1.2-3.8)	3.3 (2.4-4.4)	0.63 (0.31-1.26)	.19
OR postintervention vs preintervention (95% CI)	0.59 (0.44-0.80)	0.91 (0.78-1.06)	NA	NA	0.94 (0.64-1.37)	1.06 (0.89-1.26)	NA	NA
*P* value^b^	<.001	.23	NA	NA	.73	.49	NA	NA

Abbreviations: NA, not applicable; OR, odds ratio.

^a^ Two-way interactions were performed including preintervention vs postintervention periods and control vs intervention units for surgical units (OR for any missed dose, 0.64 [95% CI, 0.48-0.85; *P* = .002]; OR for patient refusal, 0.64 [95% CI, 0.41-1.00; *P* = .05]; and OR for reasons other than refusal, 0.65 [95% CI, 0.47-0.91; *P* = .01]) and medical units (OR for any missed dose, 0.55 [95% CI, 0.44-0.69; *P* ≤ .001]; OR for patient refusal, 0.51 [95% CI, 0.39-0.66; *P* ≤ .001]; and OR for reasons other than refusal, 0.88 [95% CI, 0.57-1.35; *P* = .56]). Three-way interactions were performed including preintervention vs postintervention periods, control vs intervention units, and surgical vs medical unit with no significant differences observed (OR for any missed dose, 0.86 [95% CI, 0.60-1.23; *P* = .41]; OR for patient refusal, 0.80 [95% CI, 0.48-1.33; *P* = .38]; and OR for reasons other than refusal, 1.35 [95% CI, 0.78-2.33; *P* = .29]).

^b^ Calculated using multiple outputation of the generalized linear mixed-effects models with the binomial family and a logit link.

No significant differences were observed between the effect of the intervention for medical vs surgical units relative to nonadministered doses for any reason (OR, 0.86; 95% CI, 0.60-1.23; *P* = .41 for interaction), patient refusal only (OR, 0.80; 95% CI, 0.48-1.33; *P* = .38 for interaction), or other reasons than patient refusal (OR, 1.35; 95% CI, 0.78-2.33; *P* = .29 for interaction). Although not significant by 3-way interaction term, the patterns for the proportion of doses not administered appeared to differ for surgical vs medical units. On surgical control units, there were significant, albeit smaller, decreases in the odds of missed doses (OR, 0.84; 95% CI, 0.74-0.95) and refused doses (OR, 0.79; 95% CI, 0.66-0.93) that were not seen on medical units (Table 3). Furthermore, a 2-way interaction performed between the preintervention vs postintervention periods and between control vs intervention units showed a difference in the patterns for the proportion of doses not administered on surgery units (OR for any missed dose, 0.64 [95% CI, 0.48-0.85; *P* = .002 for interaction]; OR for patient refusal only, 0.64 [95% CI, 0.41-1.00; *P* = .05 for interaction]; OR for other reasons than patient refusal, 0.65 [95% CI, 0.47-0.91; *P* = .01 for interaction]). A similar trend was observed on medical units (OR for any missed dose, 0.55 [95% CI, 0.44-0.69; *P* ≤ .001 for interaction]; OR for patient refusal only, 0.51 [95% CI, 0.39-0.66; *P* ≤ .001 for interaction]; OR for other reasons that patient refusal, 0.88 [95% CI, 0.57-1.35; *P* = .56 for interaction]). Three-way interactions were performed including preintervention vs postintervention periods, control vs intervention units, and surgical vs medical units with no significant differences observed for any missed dose (OR, 0.86; 95% CI, 0.60-1.23; *P* = .41), patient refusal only (OR, 0.80; 95% CI, 0.48-1.33; *P* = .38), and other reasons than refusal (OR, 1.35; 95% CI, 0.78-2.33; *P* = .29).

### Time Trend Analysis

Figure 2 shows the conditional probability of missed doses by month for the 4 strata (medical and surgical units and intervention and control groups) to show the effect over time. This analysis shows that no dramatic changes evident before the intervention period begun.

### VTE Events

The overall proportion of patients with VTE events was 0.26% in the preintervention and 0.19% in the postintervention periods (*P* = .46). The proportion of VTE events decreased from the preintervention to postintervention periods by 40.0% on intervention units (0.30% vs 0.18%; OR, 0.60; 95% CI, 0.16-2.23) and 16.6% on control units (0.24% vs 0.20%; OR, 0.81; 95% CI, 0.35-1.87). However, none of these decreases reached statistical significance.

## Discussion

We demonstrated that a patient-centered education intervention bundle delivered in a timely manner via a real-time alert significantly reduced nonadministration and patient refusal of pharmacologic VTE prophylaxis doses among hospitalized patients. We found a 43% reduction in the odds of nonadministration and a 47% reduction in patient refusal of pharmacologic VTE prophylaxis. These fewer missed doses were associated with a 40% reduction in VTE events, although with few events, this finding was not statistically significant. When we tested the effect of the intervention by unit type we found no significant differences, suggesting that the intervention was beneficial for patients on medical and surgical units. To our knowledge, this is the first quality improvement intervention to use this health information technology approach to address the critically important problem of nonadministration of VTE prophylaxis. Moreover, it efficiently targeted education just in time to patients and health care professionals and may be an effective approach to address other risks to patient safety in hospitalized patients.

Our findings are consistent with those of a small, single-center study of a pharmacist-led patient educational program, which also resulted in a significantly improved adherence to pharmacologic VTE prophylaxis.^26^ However, our study is different because we needed to complete dramatically fewer patient educational sessions. Unlike that study, our targeted intervention was delivered only to patients who have missed a dose of prophylaxis, rather than educating all patients. Targeted interventions, rather than blanket educational approaches, hold the key to addressing nonadministration of VTE prophylaxis. We efficiently focused on a relatively small group of patients (because Shermock et al^10^ previously showed that about 20% of patients account for more than 80% of all missed doses of VTE prophylaxis), a phenomenon in congruence with the Pareto principle. We did not waste time and resources educating all patients, which would be well beyond the feasibility and cost capabilities for most hospitals.

Our study is strengthened by using an education bundle that was developed collaboratively with local and national patient stakeholders and gave patients the opportunity to choose their preferred methods of education. We demonstrated the effectiveness of a patient-centered approach to health care, an aim set forth by the 2001 report by the Institute of Medicine Committee on Quality of Health Care in America.^27^ Health care systems that empower patients to make informed decisions about their health care result in improved outcomes.^28,29,30^ This study demonstrates that an intervention offering patients a choice of how to deliver health information can be efficacious.^31^ This intervention is imminently scalable because the paper and video educational materials are freely available for public use.^22^

We show this intervention to be effective regardless of admitting service, because similar declines in nonadministration were achieved on medical and surgical intervention units. The intervention reduced patient refusal of doses by nearly 50% on medical and surgical intervention units. On medical control units, there were no changes between the preintervention and postintervention periods in the odds of any missed dose, refused doses, or doses missed for other reasons. We saw a small, but significant, decrease in overall missed doses and refused doses on surgical control units. At our hospital, surgical residents routinely work on many different surgical units, whereas medical residents are primarily based on a single or limited number of medical units. This contamination may explain the small improvement observed on the surgical control units. However, rather than considering this contamination a limitation, as is usual in clinical trials, we consider it a beneficial unintended consequence, or “halo effect,” of a real-world quality improvement intervention, as previously described.^32^

This study highlights how health information technology can be harnessed to address patient care deficiencies by targeting education specific to patients in need. The Agency for Healthcare Research and Quality touts clinical decision support in the risk assessment and prescription of VTE prevention.^4,33,34^ Numerous researchers have demonstrated the role of technology in improving prescription of VTE prophylaxis.^18,19,35,36^ By alerting the health educator in real time when a medication dose was not administered, we were able to intervene in a timely fashion to engage other key people involved in phases of medication administration (ie, nurses and hospitalized patients). The targeted approach did not waste the time of nurses who were administering all doses and patients who were accepting all doses. The nurses who needed education received it in real time, and the patients were offered a patient-centered education bundle that allowed them to choose their preferred approach to learning. This approach gives patients the opportunity to make an informed decision, an important tenet of patient-centered care,^37^ and directly targets knowledge gaps among patients and their families regarding VTE.^38,39^ Wide-scale adoption of electronic health record systems, fueled by the Centers for Medicaid & Medicare Services Meaningful Use program, may truly offer the opportunity to embrace this type of solution.^40^

### Limitations

The findings of this study should be interpreted with the following limitations in mind. First, we hoped to intervene on all patients with a nonadministered dose of pharmacologic VTE prophylaxis; however, a large proportion of these patients did not receive an intervention. Notwithstanding, we demonstrate a significant decrease in the odds of nonadministration of VTE prophylaxis, using the intention-to-treat approach. If we had intervened on all patients, the magnitude of effect would have likely been greater. Second, our study was performed at a single academic medical center and may not be representative of all hospitals, limiting the generalizability of our findings. However, missed doses of VTE prophylaxis are just as common, if not more so, at community hospitals^14^ and the intervention materials are freely available for use.^22^ Third, this intervention used a robust, modifiable electronic health record system. However, the bundle could be used for patient education in response to refusal, even without an electronic alert. Fourth, nurses on these units have already been primed about the importance of VTE prophylaxis because more than 800 nurses were educated on the harms of VTE as part of an educational trial before the current study began.^41^ That trial showed a significant improvement in missed doses of VTE prophylaxis, making it harder to find an effect in the present study owing to the ceiling effect. Fifth, our study was not a randomized trial. However, we used the strongest research method possible as suggested in prior reports on quality improvement interventions.^42,43,44^ Sixth, we hired a part-time nurse educator for this intervention, which may be cost-prohibitive for other hospitals. However, we have mitigated this limitation in our ongoing dissemination project by modifying the intervention so it can be implemented using existing resources in a cost-effective manner.

## Conclusions

This report has important implications for policy makers, researchers, and health care professionals working in quality improvement to eliminate preventable harm. Before this effort, our organization scored well on quality measures that evaluated prescribing VTE prophylaxis.^45,46^ If we had not aimed for 0 preventable VTE events,^47,48^ we would not have identified that doses of VTE prophylaxis were not being administered, which offered an opportunity to further reduce harm due to VTE.^10,13^ Policy makers should ensure that the outcome and process measures are valid,^17^ avoid classifying low-quality care as high-quality, and encourage curiosity about eliminating harm.

A targeted patient-centered educational intervention deployed in a timely fashion significantly reduced nonadministration and patient refusal of pharmacologic VTE prophylaxis doses in hospitalized patients. This quality intervention improves patient engagement, awareness, knowledge, and willingness to accept optimal VTE prevention with prophylaxis. The concept behind this intervention shows promise well beyond the delivery of VTE prevention. It presents an opportunity to proactively identify when an evidence-based care practice was not delivered and intervene immediately to prevent harm, to the extent possible.

## Notes

Supplement. **Trial Protocol**
