# Supplementary material for: Effect of Real-time Patient-Centered Education Bundle on Administration of Venous Thromboembolism Prevention in Hospitalized Patients
Source: JAMA Netw Open. 2018 Nov 16;1(7):e184741. doi: 10.1001/jamanetworkopen.2018.4741 (PMC6324387; doi:10.1001/jamanetworkopen.2018.4741)
Supplement: Supplement. — Trial Protocol [file jamanetwopen-1-e184741-s001.pdf]

Title:

Education Bundle to Decrease Patient Refusal of Venous Thromboembolism Prophylaxis

Registration:

https://clinicaltrials.gov/ct2/show/NCT02402881

Funding:

Patient Centered Outcomes Institute (PCORI)

The funders will have no role in the conduct of this study

Contact Information

Elliott R. Haut, MD, PhD, FACS
Division of Acute Care Surgery, Department of Surgery
The Johns Hopkins University School of Medicine
Sheikh Zayed 6107C
1800 Orleans St.
Baltimore, MD 21287
410 502 3122 (phone)
410 502 3569 (fax)
ehaut1@jhmi.edu

1. Introduction

Public Health Impact of Venous Thromboembolism

Venous thromboembolism (VTE), comprised of deep vein thrombosis (DVT) and/or pulmonary embolism (PE), affects 350,000-600,000 individuals in the United States annually. More than 100,000 people die each year in the United States as a result of PE.[1] Numerous studies have shown that VTE prophylaxis is vastly underutilized in hospitals[2,3] and the Agency for Healthcare Research and Quality (AHRQ) has listed strategies to improve VTE prevention on its top ten list for patient safety practices.[4-7] Consequently, numerous interventions have been implemented to improve prescription of VTE prophylaxis[7-10] with the implicit assumption that medications prescribed for hospitalized patients will always be administered.

Deficits in the Administration of Prescribed Venous Thromboembolism Prophylaxis

Although the benefit of VTE prophylaxis is well established, many hospitalized patients do not receive adequate VTE prophylaxis. Studies from academic and community hospitals suggest that 10-20% of prescribed VTE prophylaxis doses are not administered, with the leading causing being patient refusal.[11-15] In order to understand practice at our own institution, we conducted an exploratory study and found that nearly 12% of prescribed doses of pharmacologic VTE prophylaxis are not administered and almost 60% of missed doses are due to patient or family member refusal.[11] In a survey of 500 recently hospitalized patients, the National Blood Clot Alliance found that only 28% and 15% respectively had basic knowledge of deep venous thrombosis (DVT) or pulmonary embolism (PE) despite the fact that 15% of participants had a history and 43% had a family history of either condition. Similarly, we found that hospitalized patients have varying understanding and preferences regarding the harms of VTE and benefits of VTE prophylaxis.[16]

Rationale for Patient-centered Education Bundle Trial

Given the high frequency of patient refusal of VTE prophylaxis and significant knowledge gaps regarding VTE, there is an urgent need to educate patients and families on the importance and benefits of compliance with VTE prophylaxis. As a part of a study funded by the Patient-Centered Outcomes Research Institute (PCORI), we first educated nurses on the harms of VTE and the benefits of prophylaxis, showing that this first step intervention improved VTE prophylaxis administration by approximately 10%.[17] The patient-centered education bundle will be delivered as an in-person, 1-on-1 discussion session with a nurse educator. Supporting education materials include a 2-page education sheet and an educational video.[18]

Study Objectives and Hypotheses

We hypothesize that patient refusal of VTE prophylaxis is associated with significant knowledge gaps among patients regarding their risk of developing VTE and the benefits

of VTE prophylaxis, and that delivering an education bundle to patients who refuse VTE prophylaxis will improve acceptance of VTE prophylaxis and decrease rates of VTE.

The objective of this study is to determine the effect of delivering a patient education intervention bundle on incidence of VTE prophylaxis non-administration, and on incidence of VTE in hospitalized patients.

Primary Hypothesis: Patients on floors in the intervention arm will have a larger decrease in frequency of missed doses of VTE prophylaxis compared with patients on floors in the control arm.

Secondary Hypothesis: Patients in the intervention arm will have a larger decrease in frequency of VTE compared with patients in the control arm.

2. Study Design

A. Study Design

- Prospective cohort study

B. Eligibility Criteria

- All patients hospitalized on 16 medical/surgical (non-ICU) floors who are prescribed pharmacologic VTE prophylaxis will be eligible for inclusion
- INTERVENTION: All patients on the four study floors (2 medical floors and 2 surgical floors) who miss at least one dose of VTE prophylaxis will be eligible to receive the patient education bundle intervention
- CONTROL: Patients on the 12 control floors (8 medical floors and 4 surgical floors) who miss doses of VTE prophylaxis will not receive the intervention
- Patients who move between floors during their hospitalization (Intervention → Control or Control → Intervention) will be excluded from analysis

C. Interventions

A patient-centered education bundle was created with input from key stakeholders including clinicians and patients. The bundle will be delivered in-person by a nurse educator. Patients may choose any one or a combination of components of the intervention including:

- In-person, 1-on-1 discussion session with the nurse educator.
- A two-page patient education paper
- Patient education video

D. Enrollment

When a dose of VTE prophylaxis is documented by the nurse as not administered in the electronic medication administration record (eMAR), a real-

time notification will be sent to the study team via pager and email. Upon receiving the alert, the nurse educator will engage the documenting bedside nurse to determine the cause for the missed dose. The nurse educator will then present the patient who missed a dose of pharmacologic VTE prophylaxis with the patient education bundle.

E. Duration of Study

The proposed intervention will be conducted over an 8-month period (April 1, 2015 through December 31, 2015).

We will use the 6-month period before the intervention (October 1, 2014 through March 31, 2015) as the pre-implementation data period.

F. Outcomes

a. Primary Outcome measure: VTE prophylaxis non-administration
b. Secondary Outcome Measures: VTE (defined by AHRQ PSI-12 diagnosis codes)

G. Data Source

Patient demographic data and VTE outcomes will be extracted from the Johns Hopkins Hospital administrative database. Pharmacologic VTE prophylaxis medication administration data will be extracted directly from electronic medical administration record (eMAR).

3. Analytic methods

A. Blinding

Our biostatistician team will be blinded to floor assignment arms.

B. Baseline Characteristics

a. Comparison of both arms to ensure similarity at baseline
b. Descriptive analysis of baseline characteristics (i.e. simple counts and proportions by trial arm)

C. Analytic Plan

Our primary hypothesis will be evaluated by comparing VTE prophylaxis non-administration before the intervention vs. during the intervention period. We will compare this change on intervention floors vs. control floors over the same period.

D. Multi-level mixed effects linear regression

Due to the complexity of the multilevel structure of the data (i.e. multiple doses per patient across various hospitalizations, nurses and floors), multiple outputation11 will be used to reduce the levels of hierarchical structure to the floor level and nurse level by randomly selecting one dosage per patient. By

reiterating the procedure 1000 times, we will estimate the odds ratios (ORs) and 95% confidence intervals conditional on the floor and nurse. For estimating conditional odds ratios and their confidence intervals, the binomial family and a logit link will be used, and for estimating the conditional proportions, the Poisson family and a log link will be used. An a priori stratified (or subgroup) analyses (medical vs. surgical floors) will be performed using the same models to assess the same outcomes. All analyses will be on an Intention-to-Treat basis (all doses and patients on each floor).

1. US Department of Health and Human Services. The surgeon general's call to action to prevent deep vein thrombosis and pulmonary embolism. http://www.ncbi.nlm.nih.gov/books/NBK44178/. Updated 2008. Accessed April 5, 2014.

2. Goldhaber SZ, Tapson VF, DVT FREE Steering Committee. A prospective registry of 5,451 patients with ultrasound-confirmed deep vein thrombosis. *The American Journal of Cardiology*. 2004;93(2):259-262.

3. Cohen AT, Tapson VF, Bergmann J, et al. Venous thromboembolism risk and prophylaxis in the acute hospital care setting (ENDORSE study): A multinational cross-sectional study. *The Lancet*. 2008;371(9610):387-394.

4. Shekelle PG, Wachter RM, Pronovost PJ, Schoelles K, McDonald KM, Dy SM, Shojania K, Reston J, Berger Z, Johnsen B, Larkin JW, Lucas S, Martinez K, Motala A, Newberry SJ, Noble M, Pfoh E, Ranji SR, Rennke S, Schmidt E, Shanman R, Sullivan N, Sun F, Tipton K, Treadwell JR, Tsou A, Vaiana ME, Weaver SJ, Wilson R, Winters BD. Making health care safer II: An updated critical analysis of the evidence for patient safety practices. . 2013;211.

5. Shekelle PG, Pronovost PJ, Wachter RM, et al. The top patient safety strategies that can be encouraged for adoption now. *Ann Intern Med*. 2013;158(5 Pt 2):365-368.

6. Haut ER, Lau BD. Prevention of venous thromboembolism: Brief update review. In: *In "making health care safer II: An updated critical analysis of the evidence for patient safety practices.* Rockville, MD.: Agency for Healthcare Research and Quality; 2013.

7. Lau BD, Haut ER. Practices to prevent venous thromboembolism: A brief review. *BMJ Qual Saf*. Mar 2014;23(3):187-195.

8. Durieux P, Nizard R, Ravaud P, Mounier N, Lepage E. A clinical decision support system for prevention of venous thromboembolism effect on physician behavior. *JAMA*. 2000;283(21):2816-21.

9. Streiff MB, Carolan H, Hobson DB, et al. Lessons from the johns hopkins multi-disciplinary venous thromboembolism (VTE) prevention collaborative. *BMJ*. 2012;344:e3935.

10. Kucher N, Koo S, Quiroz R, et al. Electronic alerts to prevent venous thromboembolism among hospitalized patients. *N Engl J Med*. 2005;352(10):969-77.

11. Shermock KM, Lau BD, Haut ER, et al. Patterns of non-administration of ordered doses of venous thromboembolism prophylaxis: Implications for novel intervention strategies. *PLoS One*. 2013;8(6):e66311.

12. Lau BD, Streiff MB, Kraus PS, et al. Missed doses of venous thromboembolism (VTE) prophylaxis at community hospitals: Cause for alarm. *J Gen Intern Med*. 2018;33(1):19-20.

13. Fanikos J, Stevens LA, Labreche M, et al. Adherence to pharmacological thromboprophylaxis orders in hospitalized patients. *Am J Med*. 2010;123(6):536-41.

14. Louis SG, Sato M, Geraci T, et al. Correlation of missed doses of enoxaparin with increased incidence of deep vein thrombosis in trauma and general surgery patients. *JAMA Surg*. 2014;149(4):365-370.

15. Haut ER, Lau BD, Kraus PS, et al. Preventability of hospital-acquired venous thromboembolism. *JAMA Surg*. 2015;150(9):912.

16. Wong A, Streiff M, Haut E, et al. Patient perspectives on pharmacological venous thromboembolism prophylaxis at the johns hopkins hospital. *J Thromb Thrombolysis*. 2013;35(3):416.

17. Lau BD, Shaffer DL, Hobson DB, et al. Effectiveness of two distinct web-based education tools for bedside nurses on medication administration practice for venous thromboembolism prevention: A randomized clinical trial. *PLoS One*. 2017;12(8):e0181664.

18. Popoola VO, Lau BD, Shihab HM, et al. Patient preferences for receiving education on venous thromboembolism prevention - A survey of stakeholder organizations. *PLoS One*. 2016;11(3):e0152084.

19. Follmann D, Proschan M, Leifer E. Multiple outputation: Inference for complex clustered data by averaging analyses from independent data. *Biometrics*. 2003;59(2):420-429.
